# Promoting Darfuri women’s psychosocial health: developing a war trauma counsellor training programme tailored to the person

**DOI:** 10.1186/1878-5085-4-10

**Published:** 2013-03-26

**Authors:** Alia Badri, Rik Crutzen, Shahla Eltayeb, HW Van den Borne

**Affiliations:** 1 Department of Health Promotion, CAPHRI School for Public Health and Primary Care, Maastricht University, Maastricht, The Netherlands; 2 School of Psychology and Pre-school Education, Ahfad University for Women, Omdurman, Sudan

**Keywords:** Darfuri women, Psychosocial war-related needs assessment, Counsellor training, Trauma counsellor characteristics, Contextual-theoretical framework, Targeted prevention, Tailored therapy

## Abstract

Women are considered special groups who are uniquely vulnerable in the context of war exposures. To effectively target the resources aimed at mitigating mental health consequences and optimising and maximising the use of mental health provisions, culturally relevant war trauma counsellor training is required. The objectives of this study are to promote a new philosophy in the Sudanese mental health care by introducing an integrative approach for targeted prevention and tailored treatments to the Darfuri person in a cost-effective way. Furthermore, the study provides evidence- and theory-based guidelines for developing a war trauma counsellor training programme in Sudan, mainly based on qualitative and quantitative studies among war-affected Darfuri female students. Cultural conceptualisations such as gender roles and religious expectations as well as theories that emphasise resilience and other psychosocial adaptation skills have been operationalised to reflect the totality of the Darfuri women’s experiences. Furthermore, the results of four interrelated studies among war-traumatised undergraduate Darfuri women who are internally displaced provide the basis that guides an outline for qualification development, capacity building and skills consolidation among Sudanese mental health care providers. Explicit war-related psychosocial needs assessment tools, specific war-related trauma counsellor training and particular counsellor characteristics, qualities and awareness that pertain to strengthening the efficacy of war trauma Sudanese counsellors are recommended. The aim is to produce expertly trained war trauma counsellors working with war-affected Darfuri women in particular and with regards to their helpfulness in responding to the psychosocial needs of war-exposed Sudanese in general.

## Review

### Introduction

Epidemiological studies have suggested that women rather than men are more likely to experience mental health consequences as a direct result of war trauma [1,2], with a two-fold greater lifetime prevalence for post-traumatic stress disorder (PTSD) than men [3,4], and demonstrate higher rates of psychiatric comorbid disorders such as depression [5,6]. Although accounting for approximately 70% of war-exposed groups [7], the tendency to marginalise women in the study of mental health care provision highlights their potential for developing future dysfunctional psychosocial capacities [8] without redress of effective psychosocial and mental health care provision. Published works that relate to the psychosocial implications of war exposures on Darfuri women and girls *per se* are scarce [9] but are crucial to effectively target the use of existing mental health provisions in Sudan.

The Ahfad University for Women (AUW) is a privately operated all women’s university in Omdurman city, Khartoum state. With a core concern to educate, safeguard and protect the future of Sudanese women and girls (AUW mission statement, 1906), notwithstanding those who have been war-traumatised, AUW is home to approximately 209 Darfuri students, an average of 3% of the total AUW student population. In order to effectively translate this mission, psychosocial and mental health services should become part and parcel of the university’s infrastructure for the relief or control of the progression of mental health illness among its internally displaced populations (IDPs). When preparing AUW war trauma counsellors, appropriate personal and professional war trauma counsellor training is required to qualify them in assuming a crucial responsible role in identifying the psychosocial needs among war survivors, especially as the trials and tribulations continue with an on-going Darfuri war that is further complicated by current on-going post-displacement stressors [10].

### Evidence-based contextual outline

In seeking to identify the psychosocial needs of Darfuri IDP war-affected women, four interrelated research studies were conducted among AUW undergraduates. Through interpretative phenomenological analysis, the narratives of 20 Darfuri students demonstrated the harrowing effects of personal and family war exposures, witnessing warfare and hearing about incidences within combat zones. Their stories illustrated an array of recurrent themes, including battles with heavy gun-fire, artillery attacks and air raids, separation and loss of family members, injury, torture and death, kidnapping, abduction and disappearance, and shortages of essential life-sustaining supplies in internally displaced camps [10].

The severity of war exposures and symptoms for PTSD was assessed through the application of a culturally relevant version of the Harvard Trauma Questionnaire (HTQ) [11] among 116 Darfuri AUW undergraduates. More than half reported being personally exposed as victims or as witnesses to war-related traumatic events with a strong association between direct war-related traumatic exposures and the full symptom of PTSD [12]. Generalised anxiety and symptoms for major depression were measured according to the Hopkins Symptom Checklist-25 (HSCL-25) [13], where 56% and 51% manifested symptoms of anxiety and depression, respectively. The most commonly reported anxiety symptoms were headaches, feeling fearful and feeling restless and tense, while depression symptoms mostly reported were self-blame, feeling blue and feeling no interest in usual activities. Anxiety scores were positively correlated with depression scores, and younger participants (15 to 20 years) who had a deceased parent were more anxious, while those whose father was a blue-collar worker (indicating low socioeconomic status) were more depressed [14].

Furthermore, evidence illustrated that they were confronted with a myriad of on-going life hassles and urban-cultural challenges. Urban-cultural challenges and lack of environmental mastery occurred to most Darfuri participants as they relocated to Omdurman city which included negotiating an unfamiliar transport system, learning the routes and directions to important city landmarks and insufficient funds for basic hygienic essentials. Also, as a result of being physically distant from their families, they lacked the shelter of parents and lost familiar and rich social support networks such as the encouragement of extended family members. The associated emotional distress patterns that emerged were similar to the symptoms of mood and anxiety disorders, according to the DSM IV criteria for symptoms of generalised anxiety disorder and major depression [14].

At a glance, there seems to be an emerging body of evidence to suggest the existence of a chain of events that in combination is associated with an increased vulnerability to psychosocial maladaptive symptoms. However, the reality is quite different. Darfuri participant’s resilience levels, as measured by The Resilience Scale [15], illustrated that 57% of the 116 student participants were between moderate to moderately low levels of resilience [14].

Coping resources and resilience characteristics were two primary aspects that influenced the mental health recovery of the Darfuri participants [16]. The strong religious practices and beliefs, availability of social support networks, reliance on making meaning (also known as meaning-attribution processes) and a positive future outlook seem to lend to their ability to cope with their subsequent emotional distress owing to war-related exposures, current on-going life hassles and urban-cultural challenges [16]. The relationship between the component parts of mental health recovery (i.e., resilience levels, protective resources and resilience characteristics) is addressed in an interactive process to help identify the psychosocial needs of Sudanese affected by war in general and Darfuris in particular, initiating a theory-based conceptual framework and thereby developing a successful trauma counsellor training programme.

### Theory-based conceptual framework

Operationalising the totality of these Darfuri women’s experiences leads to four unfolding concepts that relate to the evolving nature of the impact of location (war exposures), dislocation (displacement stressors) and relocation (coping). Concepts that incorporate these dynamic interactive processes of personal recovery are based on the theory of meaning-related processes [17,18], resilience [19,20] and positive protective social support networks [21,22].

The concept of meaning-related processes involves reappraising and attributing new meaning to unchangeable negative events that are usually beyond the control of the person [23], such as war-related traumatic exposures and post-displacement stressors and hardships. Moreover, religiosity typifies meaning attribution in that it provides a way of understanding suffering and loss [24]. The evidence has shown that IDP Darfuri women have reappraised altered and replaced internal beliefs with new meanings to fit the new circumstances of their post-displacement lives [16]. They have also used religion in the form of prayer and reading the Quran in times of severe emotional distress owing to war-related exposures, current ongoing life hassles and urban-cultural challenges [10]. Darfuris have attributed meaning that was congruent with their cultural belief systems, personal goals, commitments and motivation [25] by displaying their resilient characteristic of meaningfulness [16]. Meaningfulness of life [26] has been used to describe the extent to which reappraised new situations help war-traumatised individuals deal with and offset the negative perceptions of war-related traumatic exposures and post-displacement stressors, permitting realistic coping and a positive outcome in their adjustment process in the form of growth [27]. Operationally, Darfuris’ meaningfulness resilience characteristics gradually allowed them to become aware of the urban-cultural clashes within their new environment at AUW and Omdurman city and placed new meanings based on their cultural upbringing that allowed them to interact with others in the community [12]. Resilience connotes inner strength, competence, optimism, flexibility and the ability to cope effectively when faced with adversity [19,28]. Resilience is operationalised by cultural norms and societal expectations [29], and mobilised by internal motivation to eliminate stressful conditions, reconcile adverse experiences and achieve psychosocial adjustment in the face of adversity [30]. Evidence has shown that for many Darfuri female undergraduates, resilience seemed to be defined on the basis of meeting given by societal expectations for behavioural competence [31], and others reflected the internal and external attributes of specific cultural norms [29,32]. They seemed to demonstrate resilience by generating opportunities for success within their immediate environments [33,34] whether these are positive self-perceptions and external competences [35] or environmental mastery or academic achievements [36]. Darfuri traditional and cultural upbringing demonstrates a disposition to enter functional roles early in life where children are expected to assume productive roles and responsibilities (for example, young girls accept domestic responsibilities and take care of younger siblings). This develops and fosters an adept self-image [37], social confidence [38], prosocial behaviour [39] and self-efficacy [40,41]. Operationally, it may be possible to infer that Darfuri women trajectories from adversity to recovery through previously learnt mechanisms of hardiness and resilience play a crucial role in overcoming their psychosocial distress.

Positive social supportive networks protect and buffer against adversity and severe distress by providing active reassurance, guidance, belonging and affection. The evidence among Darfuri undergraduates seems to corroborate these concepts in the availability, range, frequency, accessibility and quality of close relationships and attachments they make in terms of alleviating psychosocial disorders [42-45]. Social support networks have created a crucial protective role in the post-displacement lives of Darfuri undergraduates such as in the absence of their customary social supports (parents, siblings, relatives, and neighbours). Darfuri women demonstrated a disposition to fall back on the habitual modes of protection from the unknown environment of Omdurman city and AUW by forming close interpersonal relationships with Darfuri students from the same tribe, ethnicity and region, and belonging to the Darfuri student association at AUW [16].

The interactive processes of the Darfuri IDP experience of war-related traumatic exposures and post-displacement stressors are important because in combination, they seem to perpetuate the association with psychiatric disorders, specifically PTSD, anxiety and depression. However, the evidence provides pointers that their protective capacities of reappraising and attributing meaning to their new circumstances, capabilities in forming new secure and positive interpersonal relationships with other Darfuri students, resilient characteristics of meaningfulness, perseverance and self-reliance and moderate resilience levels amalgamate to expand their coping repertoire and highlight the interactive healing progression from psychosocial dysfunction to psychological growth in the face of multiple adversities [16].

This interactive theoretical framework helps to explain the effective ways that Darfuri war-affected women cope with their adversities and how Sudanese women operationalise trauma and recovery, which are both important concepts to consider when planning for a culturally appropriate and targeted war trauma training programme for Sudanese trauma counsellor.

### Objectives for a sustainable Sudanese war trauma counsellor training programme

The first and foremost objective of Sudanese war trauma counsellors is to serve war-affected Sudanese as their primary advocates for what is best needed in terms of resources utilisation for the mitigation and prevention of milder mental health problems from becoming long-term mental health disorders. Furthermore, Sudanese war trauma counsellors must be equipped with the ability to recognise the mental health consequences of war trauma and post-displacement stressors faced by on-going conflict-affected Sudanese, such as Darfuri women. Understanding issues in terms of culture, tradition and norms within each sector of Sudanese society is imperative to distinguish between the possible psychosocial responses and possible coping strategies employed. Sudanese war trauma counsellors must also have the capacity to identify relevant social support systems and strengthen or enhance networks that will lend to the immediate assistance of needs and relief of war-related trauma symptoms such as PTSD, anxiety and depression. Moreover, the aim of a training programme is to professionally instruct war trauma counsellors through expert, clear, reliable and up-to-date information on culturally relevant concepts, methodologies and approaches that successfully recognise and relieve psychosocial stress, and appropriately facilitate and restore normality in the new location after displacement. Furthermore, training of Sudanese war trauma counsellors should include counsellor qualities, awareness and skills consolidation that promote existing resilience levels, resilience characteristics and coping resources [16] as well as pave the way for active implementation of newly acquired coping and adaptive strategies [46] among Sudanese war-affected IDPs.

Based on the identification of Darfuri undergraduate women’s war-related experiences and post-displacement stressors, their psychosocial needs [10] and the interpretation of resilience theories as they relate to the Darfuri totality of experiences, the following are the guidelines for the development of a comprehensive war trauma counsellor training programme targeted to strengthen the efficacy of Sudanese mental health care providers in general and build the professional capacity among AUW in-house counsellors in particular:

1. Needs assessment is the catalyst that should propel war trauma counsellors’ ability in identifying and knowing the specific Sudanese war-related psychosocial needs, vulnerabilities and risks. An AUW war trauma counsellor should be well versed on the various norms, standards and traditions of a multicultural Sudanese society as he/she considers the totality of Darfuri student experiences: as an undergraduate student, as a war survivor, as young Darfuri woman, pre-conflict aspects of Darfuri life and post-displacement stressors of Omdurman life within a systemic approach [47-49]. By clarifying the details which pertain to loss in terms of identity, relationships, support systems, roles, obligations and usual routines, the AUW war trauma counsellor identifies the loss of dignity and pride, decorum and morality, cultural and religious belief systems that the Darfuri student/client has conceptualised and operationalised as needs in their current experiences of cultural incongruence, loss of familiar social support systems and networks, financial worries and academic challenges [10]. Moreover, AUW war trauma counsellors should be able to recognise the symptoms that Darfuri student/clients are particularly unable to cope with, especially those factors relating to their pre- and post-displacement stressors, the intensity of current stressors and war-related exposures and the duration of adversity (for example, factors of separation from family and culturally determined social support systems) which illustrates their vulnerabilities and risk factors for developing mental health problems [10].

2. Recognising that during their on-going psychosocial stress of displacement, Darfuri female undergraduates’ social cognition [50] informs their perception and interpretation of social information [51] which impacts their level of functioning [12]. Further, Darfuri undergraduates’ social cognition and information processing may bear on their appraisal and reappraisal of stressors influencing their perceptions of social support with the belief that additional resources, in the form of significant others, are available [10]. AUW war trauma counsellors should be aware of Darfuri undergraduates’ operational value and their use of social engagement [52] as a positive function of seeking out the co-operation of others and as a coping mechanism against the effects of pre-and post war adversity [16]. Finally, identifying the socialisation process and all that goes with it is highly influential and operational in Darfuri lives. For example, recognising the cultural norms and tradition that make up Sudanese gender roles [53] which may inhibit Darfuri women from seeking trauma counselling is important to understand as they may act in increasing their risk and vulnerability to psychosocial dysfunction. An important pragmatic and contextual point is to implement a culturally relevant approach; for example, it is more likely that a Darfuri female student approaches and talks with other Darfuri women of the same ethnic tribe than with a man or other Sudanese woman.

3. Identification of resilience levels and resilience characteristics such as perseverance and meaningfulness or as an aspect of the environment, such as coping social support resources, are the typical types of Darfuri resilience operating to protect and buffer against adversity [14]. Improving on their healthy adaptive systems that include (a) fostering resilience levels, (b) enhancing the utilisation of effective resilient characteristics (meaningfulness, perseverance and self-reliance) and (c) promoting the successful use of protective resources (social support networks) should be the focus among AUW war trauma counsellor training programmes:

(a) Fostering resilience levels aims to enable the Darfuri war-affected female student to conceptualise herself as a resilient individual. Recognising her cultural and traditional context allows for an in-depth understanding of the issues faced and the facilitation of ownership of psychosocial responses. Helping students/clients with the skill of using words to describe emotions encourages the Darfuri student to become cognizant of how her exposure to war has impacted her mental health. Also by reinforcing habitual coping mechanisms, such as the use of prayer, their options will help mobilise resiliency and adaptation to new circumstances.

(b) Enhancing the utilisation of effective resilient characteristics encompasses an increased understanding of the mechanisms of resilience such as fostering and encouraging the pre-existing strengths. Being aware and discussing the factors/determinants of pre- and post-displacement stressors also help emphasise positive qualities in the student/client by involving her to work out a solution together with the counsellor; the counsellor could say ‘I can see that you are a great help to…’, ‘It was very brave of you to come all this way on your own’, ‘I know you are not feeling very strong right now, but you have shown a lot of strength’. Be specific, practical and realistic with advice; use phrases like ‘Would it be possible to…?’, ‘What might happen if…?’ ‘In what ways could you…?

(c) Promoting the successful use of protective resources includes identifying and knowing the availability and accessibility of potential protective factors and social support groups that exist within the community. Those can be mobilised and deployed successfully according to the needs and situations among AUW war-affected female students in particular or within the larger population of IDPs in Umbada catchment of Omdurman city. Linkages can be developed with similar groups such as peer self-help support groups, mutual emotional support initiatives and creative activities which promote problem-sharing and community involvement, thereby reducing their isolation and vulnerability.

4. Recognising and distinguishing among psychosocial disorders such as PTSD, anxiety and depression account for the main aim of trauma-focused therapies and treatment. AUW war trauma counsellors must be able to link between the traumatic event or adversity and the consequential emotions and beliefs systems. Equipped with specific training skills required for working with war-traumatised Darfuri women, AUW war trauma counsellors can challenge and modify the maladaptive behaviours of war-traumatised individuals [54,55].

5. The requirements for an effective war trauma counsellor is to be trained in the specialised methodologies, skills and approaches that successfully alleviate psychosocial stress. The following recommendations of training in specific tools can be implemented within an AUW war trauma counsellor training programme but can also be transferable to other Sudanese war trauma mental health professionals.

6. The effective use of screening instruments and mental health assessment tools as defined by the DSM IV diagnosis of PTSD, anxiety and depression can adequately identify mental health priorities, understand the psychosocial needs, and detect psychiatric symptoms. The design and/or adaptation of screening tools, such as the Trauma Screening Questionnaire [56] may also be used to mirror local idioms of emotional distress that understands the impact of social structures and belief systems.

7. Narrative Exposure Therapy (NET) is a type of exposure therapy [57,58] that was developed to treat PTSD that resulted from prolonged, severe and/or multiple exposures to war-related trauma. The efficacy of NET in under-developed countries has been demonstrated [59-62] within different cultural settings [63-65] in the treatment of war-related PTSD and comorbid major depression symptoms in children, adolescents and adults. Individual NET in combination with group-based mourning comprises an effective treatment for traumatised war survivors who have lost loved ones and have symptoms of PTSD and depression and comorbid symptoms such as social withdrawal, low self-esteem, loss of trust and feelings of guilt and shame [61]. NET, supportive counselling and psycho-education have been proven to have a positive effect with Sudanese refugees suffering from PTSD [66]. Guilt cognition reductions have also been demonstrated with the successful use of NET [67,68]. Furthermore, the feasibility to train trauma counsellors in NET has been demonstrated [69]. During a NET session, the war-traumatised Darfuri will construct a detailed chronological account of her own biography in cooperation with the trauma counsellor. The traumatic experiences would be documented by the trauma counsellor to ensure trauma focus. The grief session allows the student/client to be exposed to the feeling associated with the loss similar to the specific focus of guided mourning. At the end of the sessions, the Darfuri student should receive a written report of her autobiography. Each session lasts between 120 and 150 min for 4 weeks [65].

8. Interpersonal therapy (IPT) is aimed at the treatment of major depression among conflict and post-conflict victims in order to re-establish normal patterns of life as permanent long-term recovery goals [70]. There is evidence that IPT may be effective in reducing symptoms of posttraumatic stress and depression, either delivered individually as single gender sessions [71,72] or in a group format run in groups of three to four persons [73]. Restoration techniques can include normalisation and validation of trauma-related symptoms and distress reactions to multiple losses, enabling help-seeking behaviour and empowerment. Darfuri students/clients participants within group IPT sessions for the treatment of depression and symptoms for PTSD should have the same or similar problem areas to enable appropriate goal setting, initiate therapeutic processes and foster positive group cohesion [74]. Darfuri students that had lost parents and were coping with the grief, for example, may benefit from a discussion with other students about their emotions and be open and willing to understand their own feelings. The feasibility to train trauma counsellors in IPT has been demonstrated [75].

9. Cognitive Behavioural Techniques (CBT) operates within the meaningfulness of social interactions and interpersonal environments [76] that assimilate local and community customs. Those persons that examine the effects of war-related traumatic exposures within this context have been shown to optimise positive psychosocial outcome after traumatic exposures [77,78]. Prayer, reading of the Quran and other religious practices have also been shown to reduce subsequent development of PTSD [79] and can be viewed within the realm of relaxation techniques and vehicles to deliver CBT [80]. CBT that is offered to Darfuri undergraduate clients in a safe and understanding environment can produce psychosocial relief. In introducing CBT it may be important to reassure students/clients that its objective is not to change personal beliefs but to explore different ways of thinking about their experiences and their future enabling them to develop new considerations.

10. Guided mourning for grief, although not explicitly measured among this group of Darfuris, is it highly likely that grief reactions compounded PTSD and depression. Culturally specific grief rituals need time to develop and are important for coping with loss. Intensive dialogue must be applied to ensure effective coping.

11. Psycho-education campaigns are important for reducing potential stigma associated with seeking and sustaining. This instrument designed to support war trauma survivors can be used in screening individuals who are at risk by introducing basic understanding of mental health knowledge and providing accurate information about normal trauma responses and ways to cope [81]. Moreover, psycho-education increases general awareness and disseminates information, particularly on the rationale and function of treatments and therapies such as CBT, NET and IPT and it is necessary that both the trauma counsellor and student/client become active participants in this type of support to maintain motivation and persevere with treatment.

12. Psychological debriefing or CISD (Critical Incident Stress Debriefing) is a structured group programme, which has been widely used in disaster counselling, predominantly with adults, with positive findings. It facilitates the discussion of fears, myths and beliefs, and promotes the discharge of feelings, and empowers war-traumatised individuals to build their future [54].

13. Group therapy has a higher client-counsellor ratio which improves cost-effectiveness. They promote effective catharsis, support and a sense of identification with others, especially in bereavement, death and grief groups [82]. For women in particular, widows’ groups are very effective in organising support and help for each other.

### War trauma counsellor qualities, awareness and skills consolidation

Additionally, a war trauma counsellor must possess the personal qualities and awareness that pertain to the sensitivity of the treatment as well as be an expert on the skills and practices for needs assessment. The war trauma counsellor can ask about ordinary daily life such as friends, leisure activities, who they live with, where they live, and then go on to talk about sensitive topic such as difficulties of present life, difficulties in the past experiences of violence, displacement worries and hopes and plans for the future.

War trauma counsellors must possess the personal qualities that create and foster trust and assure confidentiality, including establishing rapport, showing interest and responding to feelings of loss, loneliness and isolation [83]. In skills consolidation, while similar to those for counsellors in general, war trauma counsellors need to adhere to best practice protocols such as active listening, listening but not forcing talk, and mirroring. This is especially important because of the potential stigma associated with mental illness and barriers to health-seeking behaviours [83]. Being aware of a Darfuri’s potential feelings of guilt, shame, hostility, anger and betrayal because of their experiences, war trauma counsellors should be empathetic, non-judgmental, and show respect and acceptance. Finally, war trauma counsellors must be aware of how they conduct themselves when faced with very distressful stories. Self-observations, monitoring and examining one’s own verbal and non-verbal communication and cues such as facial expressions, eye contact, body posture and movements and tone of voice significantly affect disclosures of very intense, sad and frightening thoughts and feelings, as well as enable effective communication and facilitate the relaxation of students/clients.

## Conclusions

Sudanese culture, in particular Darfuri tradition, continues to function within expected norms of gender roles, social commitments and cultural expectations framed within an Islamic religious perspective, where social cohesion perceptions are interpreted. The experience of war trauma and post-displacement stressors and the ensuing epistemologies of mental health can also be considered within this cultural conceptualisation of social cognitions [50]. Darfuri tradition and expected modes of behaviour emphasise resilience levels, resilient characteristics and external coping resources [16] even in the most glaring atrocities and the most severe stressors among Sudanese survivors of war trauma [84-86]. Extrapolating conceptualisations that are based on very different cultural presumptions would lose focus when considering the development of a Sudanese war trauma counsellor training programme which promotes the psychosocial health of Darfuri women.

Attention has been given to the healing process unique to the war-affected Darfuri undergraduate female. Matching evidence-based experiential components of war-related exposures and post-displacement stressors with significant psychosocial distress and symptoms for PTSD, depression and anxiety has highlighted Darfuri women’s ability to form protective social support networks, underline their moderate resilience levels and exemplify their resilient characteristics shown as perseverance and self-reliance [16]. These factors are essential when designing and implementing a war trauma counselling training programme that successfully meets the psychosocial needs of war-traumatised Sudanese.

In April 2012, Ahfad Trauma Training Treatment Center (ATTTC), funded by a grant from the United States Department of the State, was the first nationally initiated project to promote war-related trauma counselling as a comprehensive, coordinated and seamless mental health service delivery for Sudanese war survivors, including AUW students, and the Omdurman community (Umbada catchment area). It is envisaged that ATTTC will not only build on the capacities of AUW trauma counsellors, but also transfer knowledge expertise and skills acquired to other organisations and institutions to carry similar roles within their communities throughout the nation.

The initiation of this war trauma counsellors training is intended to heal the emotional and social wounds of armed conflict and discourage helplessness, disempowerment and isolation among those who seek mental health. It is envisaged that the objectives, qualities and skills proposed within this manuscript will cater for the communal coping style that is characteristic of Sudanese culture.

Participants in the first training of war trauma counsellors included AUW staff in the School of Psychology, psychologists in the military hospital, and psychiatrists in private practice throughout Omdurman and Khartoum cities. The WTF or War Trauma Foundation has provided the expertise needed to train this first batch of Sudanese war trauma counsellors based on their professional needs and working requirements. Although adept in the field of war-traumatised survivors, the 15 participants had little or no knowledge of specific concepts, methodologies and tools required for actual war trauma counselling. Further, they had limited knowledge of the personal characteristics required for effective war trauma counsellors in regards to qualities, awareness and skills consolidation.

By implementing the afore-mentioned training guidelines, it is envisaged that AUW is provided with a best value-added position in meeting its vision and mission: educating, protecting and safe-guarding women as well as restoring effective resilience characteristics, promoting helpful interpersonal relationships and facilitating the development of new coping skills. Community mental health care providers and other educational institutions or organisations who work with war-traumatised Sudanese may also benefit from the implementation of these guidelines with regards to their helpfulness, capacity building and professional development in responding to the psychosocial needs of war-exposed Sudanese.

### Recommendations

Religious, socioeconomic and other cultural influences all affect the acceptability of war-related psychosocial training and interventions. The assessment of needs and the subsequent treatment of PTSD, anxiety, depression and other comorbid symptoms should not isolate individuals from their family or the community because this will impede recovery. Research is required to better understand the perceptions of war-affected Sudanese concerning mental health care, acceptability of care, willingness to continue with treatment, and to find ways to communicate with war-affected Sudanese that validate their experiences as war survivors.

The challenge in each local situation is to validate assessment needs and adapt training guidelines that are relevant, understood and accepted by the war trauma counsellors and war-affected clients. For example, introducing culturally sensitive approaches such as CBT must be audited by the counsellors within their context to ensure optimal benefit to their communities recovering from war-related exposures [87]. Furthermore, clear aids, flexible guidelines and pragmatic manuals that successfully describe the needs of a group and predict the efficacy of treatment [88] will also facilitate the replication within other institutions, organisations and communities that require assistance in developing an effective war trauma counsellors training programme. Furthermore, according to the White Paper of the European Association for Predictive, Preventive and Personalised Medicine, health care professionals must consider the current knowledge deficit in the field and to introduce integrative approaches for targeted prevention and treatments tailored to the person [89].

## Competing interests

The authors declare that they have no competing interests.

## Authors’ contributions

AB led the study concept design, and drafting of the manuscript. RC participated in the study development and review of the manuscript. HWVDB participated in study development and design and review of the manuscript. SE participated in study concept and review of the manuscript. All authors read and approved the final manuscript.
